# Patients presenting to the hospital with MRSA pneumonia: differentiating characteristics and outcomes with empiric treatment

**DOI:** 10.1186/1471-2334-14-252

**Published:** 2014-05-10

**Authors:** Emi Minejima, Mimi Lou, Paul Nieberg, Annie Wong-Beringer

**Affiliations:** 1 University of Southern California, School of Pharmacy, 1985 Zonal Ave, Los Angeles, CA 90033, USA; 2 Huntington Hospital, 100 W California Blvd, Pasadena, CA 91105, USA

**Keywords:** Pneumonia, Methicillin-resistant *Staphylococcus aureus*, Community-onset pneumonia, Healthcare-associated pneumonia, Risk factors

## Abstract

**Background:**

Concern for MRSA in patients presented to the hospital with pneumonia may be overestimated leading to excessive prescribing of empiric anti-MRSA therapy. This study aims to identify at-risk patients and treatment outcomes.

**Methods:**

Adults hospitalized during 2005–2011 with pneumonia diagnosed within 48 h of admission were included. Medical charts were retrospectively reviewed for relevant data. Patients with MRSA were matched 1:1 to those with non-MRSA pathogen or negative culture. A published risk scoring system for MRSA pneumonia was applied.

**Results:**

268 elderly patients were included, 134 patients in each group. Compared to non-MRSA group, MRSA patients presented more acutely ill (p < 0.0001) (pneumonia severity index score, 150 vs 93; vasopressor therapy, 34% vs 6%; ICU admission, 47% vs 13%; and mechanical ventilation, 35% vs 10%) and had worse outcomes (p < 0.0001) (time to reach clinical stability, 6 vs 2.5d; length of stay, 10 vs 5d; clinical failure, 28% vs 4%; 28-day mortality, 22% vs 3%). When applied to our patients, a published risk scoring scheme had 93% sensitivity but lacked specificity at 55%; 40% of medium-risk patients did not have MRSA. A history of MRSA infection or pneumonia differentiated the latter group. Most MRSA patients (66%, 88/134) were treated empirically (primarily vancomycin) but outcome was not improved by receipt of empiric therapy.

**Conclusions:**

Use of a published risk scoring scheme with additional variables from this study can potentially reduce overprescribing of anti-MRSA empiric therapy in patients presented to the hospital with pneumonia. Prospective studies evaluating the treatment benefit of non-vancomycin alternatives as empiric therapy are needed.

## Background

Recent epidemiologic investigations indicate that the prevalence of community-associated (CA)-MRSA infections is rising, though most involve skin and soft tissue infections [1,2]. While the actual incidence of MRSA in community-acquired pneumonia remains low, the incidence of MRSA in healthcare-associated pneumonia (HCAP) accounts for 28% of healthcare-associated cases [3] and exceeds that in all pneumonia types including ventilator-associated pneumonia (VAP) [4,5]. Patients with risk factors for HCAP constitute a heterogeneous group with varying risk for drug-resistant gram-positive versus gram-negative pathogens depending on functional status and type and extent of healthcare exposure [6,7]. Concern for MRSA in patients presenting to the hospital with pneumonia may be overestimated, leading to excessive prescribing of empiric anti-MRSA therapy [5].

Shorr et al. recently published a scoring system to stratify patients presenting to the hospital with pneumonia to predict their risk for MRSA infection [8]. The risk score was developed based on a retrospective analysis of 5,975 adult patients from 62 US hospitals who had an ICD-9 code for pneumonia diagnosed within 48 h of admission of a bacterial etiology; of those 837 (14%) had MRSA pneumonia. The study cohort was split into a development and validation cohort to create the risk score. The scoring system consisted of eight variables: age <30 or >79 years, recent hospitalization, nursing home exposure within the last 90 days, prior IV antibiotic therapy within the last 30 days, ICU admission, cerebrovascular disease prior to admission, dementia, and female with diabetes; and ranged from scores of 0–10. The risk for MRSA is <10% for score of 0 or 1 compared to >30% for score of ≥6. However, the study did not evaluate outcomes associated with treatment prescribed in the different risk groups. If externally validated, this scoring system has the potential to aid clinicians in targeting empiric anti-MRSA therapy based on risk for MRSA infection.

Our study aimed to evaluate patients with community-onset pneumonia defined as patients presenting to the hospital with pneumonia with or without healthcare exposure by: 1) comparing the clinical and epidemiologic characteristics of patients with MRSA versus non-MRSA pneumonia, 2) testing the utility of Shorr’s scoring system in identifying patients at risk for MRSA, and 3) evaluating outcomes observed with empiric anti-MRSA therapy in high risk patients.

## Methods

This was a retrospective cohort study conducted at a 625-bed community-teaching hospital in Pasadena, California. The study protocol was approved by the Huntington Hospital Institutional Review Board. Patients hospitalized between January 2005 and October 2011 were screened by ICD-9 codes for pneumonia (481.X-486.X) and included if they were: 1) ≥18y, 2) diagnosed with pneumonia per CDC criteria [9] within 48 h of hospital admission, 3) had a respiratory culture taken, and 4) received ≥ 48 h of effective therapy. Patients with multiple organisms in the respiratory culture were excluded. Patients were split into two groups, those with the causative organism being MRSA vs a non-MRSA pathogen or a negative culture. Since there were many more non-MRSA pneumonia patients, those who met inclusion criteria were randomly selected to match the MRSA pneumonia patients by number. HCAP was defined per the 2005 Infectious Diseases Society of America’s hospital-acquired pneumonia (HAP), VAP and HCAP guidelines [10].

Medical charts were reviewed to obtain relevant demographic, laboratory, radiographic, and clinical information: age; comorbid conditions; prior history of pneumonia within 1 year; history of healthcare exposure or prior antibiotic exposure within 90 days per medical records or stated in the patient history; any history of MRSA infection or colonization; presence of severe sepsis requiring vasopressor support; antibiotic therapy prescribed during the admission (timing and regimen); radiographic, laboratory, and clinical progress; culture and sensitivity results; length of stay; death within 28 days of pneumonia diagnosis; and hospital readmission within 30 days of discharge. Disease severity was assessed using Acute Physiology and Chronic Health Evaluation (APACHE) II score within 24 hours of admission and Pneumonia Severity Index (PSI). The data were recorded on a structured data collection form and entered into Microsoft Access.

### Data analysis

Patients were grouped by respiratory culture results for MRSA vs non-MRSA; the latter group included those with negative cultures. Demographic and clinical variables and severity of presentation were compared to identify differentiating characteristics. Patients were stratified into high, medium, and low risk groups for MRSA using the risk scoring system by Shorr et al. [8]. Notably, history of dementia used in the scoring system was substituted in this study by presence of feeding tube and Intensive Care Unit (ICU) admission was recorded for any time during the hospital stay. Sensitivity, specificity, positive and negative predictive values were calculated to validate the risk scoring system.

The choice and timing of anti-MRSA therapy prescribed during admission was compared between groups. Outcome measures were: time to achieve clinical stability [11], clinical response at end of treatment, need for and duration of mechanical ventilation, total length of stay, 28-day mortality, and 30-day readmission. Clinical response was determined as complete if resolution or partial if improvement was noted with fever, leukocytosis, and signs and symptoms of pneumonia.

### Statistical analysis

Univariate analysis was performed using Wilcoxon rank sum tests for non-parametric continuous data or independent t-test for parametric continuous data and Fisher’s exact test and chi-square test for categorical data where appropriate using GraphPad Prism v4.0 (San Diego, CA, USA) or SAS version 9.3 (SAS Institute, Cary, NC). A p value < 0.05 denotes statistical significance. To assess for the most significant predictors for MRSA pneumonia, PSI score, residence prior to admission, presence of other infections at admission, prior antibiotic exposure within 90 days, and presence of feeding tubes were modeled in a multivariate logistic regression analysis controlling for age, APACHE II, and ≥3 comorbid conditions. In order to assess the most significant predictors of death in the MRSA pneumonia group, a multivariate logistic regression analysis controlling for APACHE II score was performed with the inclusion of the following variables: receipt of empiric anti-MRSA therapy, presence of renal dysfunction, need for mechanical ventilation, and vasopressor requirement.

## Results

### Patient characteristics and risk factors

A total of 1,615 patients with ICD-9 code for pneumonia were screened. Patients were excluded for diagnosis of pneumonia >48 h after admission (n = 128), incomplete medical record (n = 66), <48 h of antimicrobial therapy (n = 99), did not meet CDC criteria for pneumonia (n = 241), multiple organisms in the respiratory culture (n = 482) or none taken (n = 288). A total of 311 patients met inclusion criteria; 134 patients had MRSA pneumonia. An equal number of patients with non-MRSA pneumonia were randomly selected to match the MRSA group by number. Twenty-eight patients in the non-MRSA pneumonia group had a bacterial etiology: *P. aeruginosa* (n = 7), *Enterobacter spp* (n = 3), methicillin-sensitive *S. aureus* (n = 3), *S. pneumoniae* (n = 3), *Acinetobacter spp* (n = 2), *S. pyogenes* (n = 2), *Haemophilus spp*. (n = 1), *Stenotrophomonas maltophilia* (n = 1), *Legionella* (n = 1).

Overall, the study cohort was elderly with a median age of 79 y and 48% were male. The most common comorbid condition was cardiovascular disease (66%), followed by pulmonary diseases (32%). Patients with MRSA vs non-MRSA pneumonia had distinct demographic and clinical characteristics compared to those with non-MRSA pneumonia (Table 1). MRSA patients were more likely to have ≥3 comorbid conditions (64% vs 30%, p < 0.0001), diabetes (33% vs 19%, p < 0.0001), cerebral vascular accident (CVA) (31% vs 7%, p < 0.0001), and renal disease (16% vs 6%, p = 0.011) than patients with non-MRSA pneumonia. Notably, majority of the MRSA patients (73% vs 17%, p < 0.0001) resided in a skilled nursing facility (SNF) prior to admission and half had enteral feeding tubes.

**Table 1:** Demographics and patient characteristics

**Characteristics**	**MRSA pneumonia n = 134 (%)**	**Non-MRSA pneumonia n = 134 (%)**	**p value**
**Demographics**			
Age [median (IQR)]	82 (71.5, 89)	75.5 (62, 85)	0.0002
Male	67 (50)	62 (46)	0.62
Residence prior to admission			
Home	35 (26)	110 (82)	< 0.0001
SNF	98 (73)	23 (17)	< 0.0001
Other hospital	1 (0.7)	1 (0.7)	1
APACHE II [median (IQR)]	15 (11,20)	10 (7,13)	<0.0001
**Comorbid conditions**			
Cardiovascular disease^a^	92 (69)	84 (63)	0.37
Diabetes	44 (33)	26 (19)	<0.0001
Cerebrovascular accident (CVA)	41 (31)	9 (7)	<0.0001
Liver disease^b^	6 (4)	4 (3)	0.74
Renal disease^c^	22 (16)	8 (6)	0.011
Pulmonary disease^d^	47 (35)	39 (29)	0.24
Malignancy	21 (16)	14 (10)	0.37
≥3 comorbid conditions	86 (64)	40 (30)	<0.0001
**Healthcare exposure history**			
History of MRSA infection	27 (20)	1 (0.7)	<0.0001
History of MRSA pneumonia	14 (10)	0	<0.0001
Prior healthcare exposure (within 90 days)	120 (90)	50 (37)	<0.0001
Prior antibiotic exposure (within 90 days)	70 (52)	23 (17)	<0.0001
Days since last antibiotic given [median (IQR)]	24 (7,41)	7 (0,32)	0.629
Prior history of pneumonia within 1 year	56 (42)	16 (12)	<0.0001
Presence of feeding tube	67 (50)	15 (11)	<0.0001
Presence of feeding tube plus history of CVA	26 (19)	4 (3)	<0.0001
**Clinical presentation**			
Presence of other infections on admission	55 (41)	13 (10)	<0.0001
Admission of intensive care units	63 (47)	18 (13)	<0.0001
Sepsis requiring vasopressor support	45 (34)	8 (6)	<0.0001
Need for mechanical ventilation	47 (35)	14 (10)	<0.0001
PSI class IV or V	123 (92)	71 (53)	<0.0001

NOTE: SNF = skilled nursing facility; PSI = Pneumonia Severity Index; APACHE = Acute Physiology and Chronic Health Evaluation; IQR = interquartile range; ^a^Hypertension, congestive heart failure, prior myocardial infarction, coronary artery disease; ^b^Total bilirubin >2.5 mg/dL; ^c^Scr >2, Chronic Kidney Disease stage 3 or worse; ^d^Chronic obstructive pulmonary disease, emphysema, asthma.

Fisher’s exact or Chi-square test done for categorical variables; p value < 0.05 denotes significance.

Wilcoxon-Mann–Whitney test done for continuous variables, p value < 0.05 denotes significance.

Healthcare exposure within 90 days and prior antibiotic exposure were significantly more prevalent in the MRSA than the non-MRSA group: 90% vs 37% and 52% vs 17%, respectively (p < 0.0001). Furthermore, prior history of pneumonia within 1 year of admission (42% vs 12%) and a history of MRSA infection (20% vs 0.7%, p < 0.0001; predominantly pneumonia) were almost exclusively present in the MRSA group. Notably, 41% of the MRSA group had other infections at the time of admission compared to 10% in the non-MRSA group. When the above risk factors were included in a multivariate regression model, residence in a SNF prior to admission (OR 5.30, CI 2.59-10.87) and prior antibiotic exposure within 90 days (OR 4.62, CI 2.16-9.91) were the two most significant predictors of MRSA pneumonia (Table 2).

**Table 2:** Risk factors predictive of MRSA pneumonia by multivariate logistic regression

**Variable**	**OR (95% CI)**	**p-value**
Age	0.995 (0.970 - 1.021)	0.7119
APACHE II	1.035 (0.972 - 1.103)	0.2833
≥3 comorbid conditions	1.947 (0.975 - 3.888)	0.0588
PSI IV & V	3.827 (1.260 - 11.626)	0.0179
Residence in SNF prior to admission	5.301 (2.585 - 10.870)	<0.0001
Presence of other infections at admission	3.686 (1.515 - 8.967)	0.0040
Prior antibiotic exposure within 90 days	4.624 (2.158 - 9.909)	<0.0001
Presence of feeding tubes	2.357 (1.062 - 5.231)	0.0350

NOTE: PSI = Pneumonia Severity Index; APACHE = Acute Physiology and Chronic Health Evaluation.

MRSA pneumonia patients presented more acutely ill compared to the non-MRSA group (p < 0.0001) as evident by the higher APACHE II scores and more patients in PSI class IV and V and admitted to the ICU with need for vasopressor therapy and mechanical ventilation.

### Utility of Shorr’s risk score

When patients were stratified by Shorr et al’s risk scoring scheme, 55% of the patients with non-MRSA pneumonia were deemed to have low risk (scores 0–1) compared to 93% of MRSA patients to have medium (64%, scores 2–5) or high risk (29%, scores 6–10). A risk score of >1 (medium-high risk) had a 93% sensitivity but lacked specificity (55%). The negative and positive predictive values were 89% and 68%, respectively. Notably, 40% of the patients with a medium risk score did not have MRSA pneumonia. Additional variables that significantly differentiated the medium-risk patients with MRSA versus non-MRSA infection were: APACHE II score, PSI class IV and V, need for vasopressor therapy, presence of other infections at admission, history of MRSA infection, and prior history of pneumonia within 1 year of admission (Table 3).

**Table 3:** Clinical characteristics, empiric treatment, and outcomes of patients stratified by risk for MRSA

	**Stratification by Shorr risk scoring scheme**								
	**Low risk (0–1)**			**Medium risk (2–5)**			**High risk (6–10)**		
	**MRSA n = 9 (%)**	**Non-MRSA n = 74 (%)**	**P value**	**MRSA n = 86 (%)**	**Non-MRSA n = 58 (%)**	**P value**	**MRSA n = 39 (%)**	**Non-MRSA n = 2 (%)**	**P value**
**Variables used in Shorr’s risk scoring scheme**									
Age [median (IQR^a^)]	66 (61,76)	74.5 (61,84.3)	0.42	84 (72.8, 89.3)	77 (63.5,85.3)	0.01	82 (76,93)	85.5 (82,89)	0.7
Recent hospitalization within 90 d	0	0		27 (31)	26 (45)	0.12	35 (90)	1 (50)	0.23
Prior antibiotic exposure in last 30 d	0	2 (3)	1	20 (23)	8 (14)	0.2	22 (56)	1 (50)	1
SNF	5 (56)	3 (4)	0.0002	63 (73)	18 (31)	<0.0001	30 (77)	2 (100)	1
Admission into ICU	0	0		34 (40)	16 (28)	0.16	29 (74)	2 (100)	1
History of CVA	0	1 (1)	1	23 (27)	8 (14)	0.1	18 (46)	1 (50)	1
Female with diabetes	1 (11)	3 (4)	0.37	15 (17)	9 (16)	0.82	9 (23)	0	0.07
**Additional variables identified in this study (not included in Shorr’s risk scoring scheme)**									
APACHE II [median (IQR)]	11 (8,18.5)	9 (7,11)	0.1	15 (11, 19.3)	12 (8,17)	0.006	18 (13,22)	21.5 (21,22)	0.52
PSI IV and V	8 (89)	31 (42)	0.01	78 (91)	38 (66)	0.0004	37 (95)	2 (100)	1
Vasopressor	0	0		25 (29)	6 (10)	0.0075	20 (51)	2 (100)	0.49
Mechanical ventilation	1 (11)	0	0.11	27 (31)	13 (22)	0.26	19 (49)	1 (50)	1
Prior antibiotic exposure in last 90 d	1 (11)	3 (4)	0.37	29 (34)	19 (33)	1	35 (90)	1 (50)	0.23
History of MRSA infection	1 (11)	1 (1)	0.2	19 (22)	0	<0.0001	7 (18)	0	1
History of pneumonia	1 (11)	5 (7)	0.5	33 (38)	10 (17)	0.009	22 (56)	1 (50)	1
Presence of other infections at admission	1 (11)	1 (1)	0.2	36 (42)	11 (19)	0.006	18 (46)	1 (50)	1
Presence of feeding tube	0	0		32 (37)	13 (22)	0.07	34 (87)	2 (100)	1
**Treatment**									
Empiric anti-MRSA therapy	3 (33)	15 (20)	0.4	58 (67)	22 (38)	0.0006	27 (69)	1 (50)	0.54
Vancomycin	2 (67)	15 (100)	0.17	53 (62)	20 (91)	0.002	21 (54)	1 (100)	1
Other agents	1 (33)	0		5 (6)	2 (9)	0.7	6 (15)	0	1
**Outcomes**									
Time to stability [median days (IQR)]	4 (3,11)	1 (1,4)	0.03	6 (4,10)	4 (1,7)	0.052	6.5 (5.3,12.8)	9.5 (0,19)	#
Clinical failure	1 (11)	0	0.11	23 (27)	5 (9)	0.0092	14 (36)	0	0.54
Mortality 28-day	0	0		18 (21)	4 (7)	0.03	11 (28)	0	1
LOS^b^ survivors [median days (IQR)]	9.5 (7,15)	4.5 (3,7)	0.0005	10 (7,14)	7 (5,14.3)	0.1	10 (6,17)	12.5 (5,20)	0.93
Readmission 30-day	2 (22)	3 (4)	0.09	10 (12)	9 (16)	0.65	6 (15)	0	1

NOTE: ^a^IQR = interquartile range; ^b^LOS = length of stay; CVA = cerebral vascular accident; SNF = skilled nursing facility; PSI = Pneumonia Severity Index; APACHE = Acute Physiology and Chronic Health Evaluation; # n too small.

Chi-square test done for categorical variables; p value < 0.05 denotes significance.

Wilcoxon-Mann–Whitney test done for all continuous variables; p value < 0.05 denotes significance.

### Treatment

Sixty six percent (88/134) of the MRSA pneumonia group received empiric anti-MRSA therapy, most (85%, 75/88) by 8 h, 11% (10/88) by 24 h, and 3% (3/88) by 48 h of admission (Table 3). Vancomycin was the most frequently prescribed agent (60%, 53/88) for the entire treatment duration; 18 patients switched from vancomycin to linezolid 96 hours after the start of therapy while 14 patients had linezolid as primary therapy. (data not shown) Patients without empiric anti-MRSA therapy had a median delay of 3 days (IQR 2, 5) before receiving anti-MRSA therapy.

Notably, 28% (38/134) of non-MRSA patients were also prescribed empiric anti-MRSA therapy. Interestingly, 97% (37/38) of these patients were classified as having low (15/38) or medium risk (22/38) of MRSA pneumonia per Shorr’s scoring scheme. Overprescribing of anti-MRSA therapy in non-MRSA patients could potentially be reduced from 28% to 3% if either history of MRSA infection or prior history of pneumonia within 1 year of admission is applied.

### Outcomes

Patients with MRSA pneumonia had significantly worse outcomes overall: prolonged time to reach clinical stability (6d vs 2.5d) and length of stay (10d vs 5d) as well as lower rates of clinical response (67% vs 97%) and higher 28-day mortality (22% vs 3%) (all p < 0.0001) (Table 3). Those classified as having low risk for MRSA (n = 83) had the most favorable outcome compared to those having medium (n = 144) and high (n = 41) risk, with increasing rates of clinical failure (1% vs 19% vs 34%), 28-day mortality (0% vs 15% vs 27%) and 30-day readmission (6% vs 13% vs 15%) respectively (Table 3). Time to reach clinical stability (2d vs 5d vs 7d) and length of stay for survivors (5d vs 9d vs 10d) also followed the same trend when comparing low, medium and high risk patients.

Patients with MRSA pneumonia were more likely to have received empiric anti-MRSA therapy (n = 88) if they were sicker at presentation with higher APACHE II (18 vs 13; p < 0.0001), PSI scores (153.4 vs 131.3, p = 0.0011), ICU admission (55%, 48/88 vs 33%, 15/46, p = 0.02), and need for vasopressor therapy (42%, 37/88 vs 17%, 8/46, p = 0.0041); and if they were from a SNF (82%, 72/88 vs 57%, 26/46, p = 0.0043). Despite receipt of empiric therapy in this sicker cohort, worse outcomes were observed with a higher rate of clinical failure (34% vs 17%, p = 0.046) and 28-day mortality (27% vs 11%, p = 0.29) (Table 4). The potential benefit of empiric treatment on outcome may have been confounded by severity of pneumonia presentation. In a subgroup analysis of the sicker patients with severity scores greater than the mean of APACHE II score ≥18 and PSI score ≥153, outcomes did not differ with respect to length of stay, the rate of clinical failure and mortality but 30-day readmission rate was significantly higher in the delayed treatment group (50% vs 6%, p = 0.02). By multivariate analysis controlling for underlying severity of illness as measured by APACHE II score, those who received empiric therapy had a 1.6 fold higher risk of death compared to directed therapy, although not statistically significant (p = 0.45) (Table 5). The need for vasopressor was the most significant risk factor predictive of death (OR 3.602, p = 0.037).

**Table 4:** Comparison of patient characteristics and outcomes of those with MRSA pneumonia who received empiric vs directed anti-MRSA therapy

**Characteristics**	**Empiric n = 88 (%)**	**Directed n = 46 (%)**	**p value**
Age [median (IQR^a^)]*	81 (72.3,88)	85.5 (69.8,93)	0.35
Residence prior to admission			
Home	15 (17)	20 (43)	0.004
SNF	72 (82)	26 (57)	0.004
APACHE II score [mean, SD^b^]^Y^	17.9 ± 6.9	13.1 ± 5.2	<0.0001
PSI score [mean, SD]^Y^	153.4 ± 36.82	131.3 ± 34.9	0.0011
Mechanical ventilation	33 (38)	14 (30)	0.45
Duration of mechanical ventilation [median IQR]*	3 (2,7)	6 (2.5,20.75)	0.064
Vasopressor use	37 (42)	8 (17)	0.0041
Outcomes			
Time to reach clinical stability [median (IQR)]*	7 (4,10)	6 (3.5,11.5)	0.47
Clinical response	55 (63)	35 (76)	0.12
Mortality at 28 days	24 (27)	5 (11)	0.029
Readmission within 30 days	11 (17)	7 (18)	0.79
Length of hospital stay of survivors [median (IQR)]*	10 (6,15)	8.5 (7,14.3)	0.50

NOTE: ^a^IQR = interquartile range; ^b^SD = standard deviation; SNF = skilled nursing facility; PSI = Pneumonia Severity Index; APACHE = Acute Physiology and Chronic Health Evaluation.

Chi-square test done for categorical variables; p value < 0.05 denotes significance.

*Wilcoxon-Mann-Whitney test, p value < 0.05 denotes significance.

^Y^Student’s t-test, p value < 0.05 denotes significance.

**Table 5:** Risk factors predictive of 28d mortality in patients with MRSA pneumonia by multivariate logistic regression

	**OR (95% CI)**	**p value**
Receipt of empiric anti-MRSA therapy	1.634 (0.461 - 5.793)	0.4468
Renal dysfunction	2.797 (0.838 - 9.343)	0.0946
Mechanical ventilation	2.747 (0.941 - 8.024)	0.0646
Vasopressor requirement	3.602 (1.078 - 12.039)	0.0374
APACHE II score	1.087 (0.987 - 1.198)	0.0903

NOTE: APACHE = Acute Physiology and Chronic Health Evaluation.

Among those with MRSA pneumonia and classified as “high risk” (n = 39), empiric anti-MRSA therapy did not reduce 28-day mortality, clinical failure, and readmission rates. However, without empiric therapy, patients had longer hospital stay (17d vs 9d, p = 0.03) and time to reach clinical stability (12.5d vs 6d, p = 0.1). Vancomycin was the primary agent prescribed empirically in over half of those patients (21/39). The number of remaining patients who were initiated with or switched to linezolid was too small to allow for meaningful comparisons.

## Discussion

Concerns for the spread of CA-MRSA have led to widespread prescribing of empiric anti-MRSA therapy for patients presented to the hospital with pneumonia. Therefore our study aimed to identify differentiating characteristics for MRSA pneumonia and evaluate outcomes associated with empiric anti-MRSA therapy. Our study cohort involved an elderly population who differed significantly between those with MRSA vs non-MRSA pneumonia on risk factors, severity of presentation, and outcomes. Jung et al. published a retrospective study of community-onset MRSA vs non-MRSA pneumonia in a similar patient population and found comparable rates of mortality and length of stay, though antibiotic treatment was not evaluated [12].

Shorr et al. recently published a risk score assessment to aid clinicians in identifying those at risk for MRSA among patients presenting to the hospital with pneumonia. When applied to our study cohort, the scoring scheme had a positive predictive value of 68%, predicting those with non-MRSA pneumonia to have low risk and those with MRSA pneumonia to have high risk reasonably well. However, majority of patients fell within the medium risk group. Applying additional differentiating criteria identified from our study (e.g. history of MRSA infection or history of pneumonia within 1 year) reduced the number of non-MRSA patients in the medium risk group from 43% to 7%. Also, addition of the PSI class to the scoring scheme helped capture the small number of patients with MRSA pneumonia who were “falsely” deemed to have low risk as 89% of them had PSI IV or V compared to 42% in the non-MRSA group.

The 2005 Infectious Diseases Society of America guideline definition of HCAP has been criticized for its lack of specificity in identifying risks for resistant pathogens [8], leading to overprescription of broad-spectrum antibiotics [6,12,13]. Furthermore, a study reported that guideline-adherent therapy in the management of nosocomial pneumonia, including MRSA HCAP was associated with increased mortality, hypothesized to be due to drug toxicities from aminoglycoside and colistin [13]. Over one-third (37%) of the non-MRSA pneumonia patients were identified as HCAP by definition and 28% were given anti-MRSA therapy. By applying Shorr’s scoring scheme, unnecessary prescribing of anti-MRSA therapy in low risk patients without MRSA pneumonia could have been reduced by 20%. Among the 11% of “low risk” patients who had MRSA pneumonia in our cohort, outcomes were similar regardless of receipt of empiric anti-MRSA therapy.

Vancomycin was the most commonly prescribed anti-MRSA therapy. Although 90% of MRSA pneumonia patients fit the definition of HCAP, the initiation rate of empiric anti-MRSA therapy was suboptimal at 68%, underscoring the need to improve early recognition of at-risk patients. By using the scoring system to guide empiric therapy, an additional 30% (40/134) of medium or high risk patients with MRSA pneumonia would have been promptly started on effective therapy. However, it is notable that a delay of 3 days in initiating anti-MRSA therapy did not lead to negative outcomes even among the subgroup of critically ill patients (APACHE II ≥18) with more severe presentation (n = 37), although, the number of patients in this subgroup is small and most received vancomycin therapy. It is possible that empiric therapy with non-vancomycin alternatives (ie. linezolid or ceftaroline) may alter outcomes in this subgroup and therefore deserves to be studied further.

### Limitations

This study has several limitations. First, our findings may not be applicable to all settings or patient populations due to the retrospective, single center design. We were unable to control for potentially confounding variables on outcomes such as antibiotic dosing and management of concurrent infections. MRSA grown from the respiratory culture may not represent the causative pathogen; however, we attempted to limit this possibility by including only patients who had MRSA as the sole pathogen from culture.

We included culture negative patients in the non-MRSA group in order to capture all those who presented to the hospital with pneumonia and received antibacterials for greater than 48 hours. Of the 106 patients who were culture negative in the non-MRSA group, 27/106 (25%) received empiric anti-MRSA therapy with the median duration of 4 days (IQR 1, 5.25). Outcomes were similar between those with negative cultures who received empiric anti-MRSA (n = 27) vs those did not (n = 79): favorable response in 25/27 (93%) vs 77/79 (97%), length of hospital stay (median: 6 vs 5 days). This data confirms that MRSA was not a likely pathogen in the culture-negative cohort and that anti-MRSA therapy was not needed in that group.

The definition of pneumonia used in this study was the CDC definition published in 2008. After our study had been completed, an updated definition was published in January 2014. Since the clinically defined pneumonia criteria remained the same and our first inclusion criteria was diagnosis of pneumonia regardless of culture results, we believe our screening criteria for inclusion into the study remains applicable. We acknowledge that the use of ICD-9 codes to identify patients can be inaccurate due to coding bias; however, we have attempted to rectify this limitation by including only patients who have met the clinical definition of pneumonia per the CDC criteria. Lastly, readmission rates could not be captured consistently as we do not have a closed healthcare system in which patients may be readmitted to the other institutions.

## Conclusions

Utilization of the HCAP definition to determine patients at risk for MRSA infection leads to excessive prescribing of anti-MRSA antibiotics. The Shorr’s risk scoring scheme improves identification of patients at high risk and low risk for MRSA pneumonia presenting to the hospital; however, 22% deemed to be at medium risk did not have MRSA. Based on findings from our elderly cohort, inclusion of additional variables of history of pneumonia within 1 year of admission, history of MRSA infection, and high PSI score strengthen the utility of this new scoring system. MRSA pneumonia patients present more acutely ill and have worse outcomes compared to their non-MRSA cohort. Receipt of empiric anti-MRSA therapy, primarily vancomycin, did not improve outcomes for patients with MRSA pneumonia. Prospective studies evaluating the treatment benefit of non-vancomycin alternatives as empiric therapy are needed.

## Competing interests

This study was supported by an investigator-initiated research grant from Forest Pharmaceuticals to Dr. Annie Wong-Beringer.

## Authors’ contributions

EM contributed to design, acquisition of data, analysis and interpretation of data; drafted the submitted article; and provided final approval of the version to be published. MS contributed to analysis and interpretation of data; critically revised the submitted article; and provided final approval of the version to be published. PN contributed to the design of the study; critically revised the submitted article; and provided final approval of the version to be published. AWB contributed to conception and design; to analysis and interpretation of data; drafted the submitted article; and provided final approval of the version to be published.

## Pre-publication history

The pre-publication history for this paper can be accessed here:

http://www.biomedcentral.com/1471-2334/14/252/prepub
